# Effectiveness of Combining Compensatory Cognitive Training and Vocational Intervention vs. Treatment as Usual on Return to Work Following Mild-to-Moderate Traumatic Brain Injury: Interim Analysis at 3 and 6 Month Follow-Up

**DOI:** 10.3389/fneur.2020.561400

**Published:** 2020-11-10

**Authors:** Emilie Isager Howe, Silje C. R. Fure, Marianne Løvstad, Heidi Enehaug, Kjersti Sagstad, Torgeir Hellstrøm, Cathrine Brunborg, Cecilie Røe, Tonje Haug Nordenmark, Helene L. Søberg, Elizabeth Twamley, Juan Lu, Nada Andelic

**Affiliations:** ^1^Department of Physical Medicine and Rehabilitation, Oslo University Hospital, Oslo, Norway; ^2^Institute of Clinical Medicine, Faculty of Medicine, University of Oslo, Oslo, Norway; ^3^Research Centre for Habilitation and Rehabilitation Models and Services (CHARM), Institute of Health and Society, University of Oslo, Oslo, Norway; ^4^Department of Research, Sunnaas Rehabilitation Hospital Trust, Nesoddtangen, Norway; ^5^Department of Psychology, University of Oslo, Oslo, Norway; ^6^The Work Research Institute, Oslo Metropolitan University, Oslo, Norway; ^7^Department of Vocational Rehabilitation, Norwegian Labour and Welfare Administration, Oslo, Norway; ^8^Oslo Centre for Biostatistics and Epidemiology, Research Support Services, Oslo University Hospital, Oslo, Norway; ^9^Faculty of Health Sciences, Oslo Metropolitan University, Oslo, Norway; ^10^Center of Excellence for Stress and Mental Health, Veterans Affairs (VA) San Diego Healthcare System, San Diego, CA, United States; ^11^Department of Psychiatry, University of California, San Diego, La Jolla, CA, United States; ^12^Division of Epidemiology, Department of Family Medicine and Population Health, Virginia Commonwealth University, Richmond, VA, United States

**Keywords:** traumatic brain injury, randomized controlled trial, return to work, cognitive remediation, vocational rehabilitation

## Abstract

**Aims:** Knowledge regarding the most effective return to work (RTW) approaches after traumatic brain injury (TBI) is lacking. This trial aimed to compare the effectiveness of a combined cognitive and vocational intervention to treatment as usual (TAU) on RTW and work stability after TBI.

**Methods:** We performed a parallel-group randomized controlled trial (RCT) at a TBI outpatient clinic at Oslo University Hospital (OUH), Norway. Patients with a history of mild-to-moderate TBI (*n* = 116) aged 18–60 were randomized (1:1) by an independent investigator to receive group-based compensatory cognitive training (CCT) and supported employment (SE) (*n* = 60) or TAU consisting of individualized multidisciplinary treatment (*n* = 56). Participants were enrolled 2–3 months post-injury. The nature of the intervention prevented blinding of patients and therapists, however, outcome assessors were blinded to group allocation. The primary outcome measure was RTW at 3 and 6 months following study inclusion. Secondary outcomes were work percentage, stability, and productivity. The present study provides results from an interim analysis from the first two planned follow ups, while subsequent publications will present results up to 12 months following study inclusion.

**Results:** Mixed effects models showed no between-group differences in the RTW proportion, work percentage, and hours worked between CCT-SE and TAU from baseline to 6 months. A significantly higher proportion of participants in CCT-SE had returned to work at 3 months when adjusting for baseline differences. The majority of participants who were employed at 3 and 6 months were stably employed. There was a statistically significant within-group improvement on RTW proportion, hours worked and work percentage in both groups.

**Conclusion:** The results revealed no difference between CCT-SE and TAU on work-related outcomes from baseline to 6 months. However, there was a higher RTW proportion in the CCT-SE group compared to TAU at 3 months. Future publications will assess the effectiveness of CCT-SE vs. TAU up to 12 months.

**Clinical Trial Registration:** US National Institutes of Health ClinicalTrials.gov, identifier #NCT03092713.

## Introduction

Failure to return to work (RTW) and decreased work stability following traumatic brain injury (TBI) constitutes a major personal and societal burden (1). A substantial proportion of those who sustain a TBI are of working age (2), and the lifetime work loss costs of TBI in the US have been estimated to almost $70 billion (3), while costs due to productivity loss and early retirement have been estimated to approximately €19 billion annually in Europe (4). Work participation is not only important financially, but is also related to quality of life, self-esteem, and social interaction (5). Thus, improving employment participation post-TBI is a critical goal in rehabilitation programs for patients with a history of TBI.

To resume and maintain employment while experiencing post-injury difficulties is challenging for many individuals with TBI (6). Overall, it is estimated that approximately 40% are able to RTW 1 year after injury (7). Most patients with mild TBI (mTBI) resume work within weeks to months after their injury (8). Still, between 5 and 30% of individuals who sustain a mild or moderate TBI are unable to RTW within 6–12 months (8, 9). Considering that approximately 70–90% of all TBIs are classified as mild (1), this represents a substantial number of people. Studies have also shown that individuals who resume employment may continue to experience symptoms affecting work stability and productivity (10–13).

In an effort to identify individuals at risk of adverse vocational outcome after TBI, several studies have assessed individual characteristics associated with reduced likelihood of resuming employment. Among the most consistently linked factors are age, education, pre-injury employment status, duration of post-traumatic amnesia (PTA) and hospital admission, extracranial injuries, functional level, emotional and cognitive status, and access to social support (14–17). Although injury-related factors seem to be influential early on, psychological distress, maladaptive coping style and lack of social support may be of greater importance in the longer term (18–21).

Cognitive deficits are common after TBI and have consistently been linked to negative employment outcome (15, 22, 23). Cognitive skills such as learning new tasks, and social interaction at the workplace are crucial for job performance. A literature review by Mani and colleagues (22) found that executive functioning, attention, memory, and verbal skills were predictive of RTW post-TBI. The review also found evidence for the efficacy of cognitive rehabilitation in facilitating RTW. In later years, researchers have urged increased attention to modifiable factors such as cognitive and psychosocial sequelae to tailor vocational rehabilitation programs and maximize outcome (11).

Although individual and injury related characteristics associated with RTW have been extensively studied, the impact of work-place factors has not attracted comparable attention (21). One study found greater independence and decision-making latitude at work to be predictive of higher RTW rates for patients with mTBI (24). A qualitative study involving 12 individuals with mTBI reported more positive experiences with RTW in workplaces with a supportive work culture (25). Cancelliere et al. (8) performed a synthesis of systematic reviews on factors affecting RTW after injury and illness. They found support for involving multiple stakeholders (i.e., employee, employer, health care providers, and employment service providers), work accommodation, multidisciplinary interventions, and return-to-work coordination. These stated factors are in line with recommendations by Wehman et al. (26) stating that communication and collaboration between stakeholders, in addition to workplace support, is essential in promoting successful RTW.

Previous interventions aimed at returning individuals to competitive employment after mild or moderate TBI have focused mainly on providing information and advice (27) or trying to increase work participation through alleviation of post-concussive symptoms (28–30). For example, Man et al. (30) assessed the efficacy of virtual reality-based training vs. a psycho-educational program in a civilian sample with mild-to-moderate TBI, and found no significant differences in vocational outcome between the two groups. Vikane et al. (29) evaluated the effect of multidisciplinary follow-up program vs. follow-up by general practitioners (GPs) for patients with persistent symptoms 2 months after mTBI. The multidisciplinary program reduced the number of symptoms, but there was no difference between the groups regarding RTW. Scheenen et al. (28) compared cognitive behavioral therapy to telephonic counseling in a civilian sample 4–6 weeks post mTBI. Results showed no significant difference regarding RTW, but surprisingly indicated that the patients receiving telephone counseling had more favorable outcome as measured by Glasgow Outcome Scale-Extended and fewer post-traumatic complaints. In summary, the existing literature is equivocal and does not provide strong clinical recommendations regarding vocational rehabilitation for people with longstanding post-concussive symptoms.

A few clinical trials exploring the effect of combining cognitive rehabilitation efforts with vocational support have been developed over the past few years. Twamley et al. (31, 32) performed a randomized controlled trial (RCT) comparing a 12-week compensatory cognitive training (CogSMART) and supported employment (SE) intervention to enhanced SE for unemployed veterans with mild-to-moderate TBI. CogSMART included strategies to improve sleep, fatigue, headaches and tension, and compensatory cognitive strategies for prospective memory, attention, learning and memory, and executive functioning. The duration of SE, which was delivered according to the principles of Individual Placement and Support (33) was 12 months. The findings suggested that the intervention improved quality of life, symptom levels and prospective memory, and speeded RTW, but there were no differences regarding RTW over the long term.

A Campbell review (34) evaluated the effectiveness of vocational interventions in individuals with TBI and concluded that there is a need for more RCTs that assess a broader range of employment outcomes, including studies of adult civilian populations outside the US. Hence, well-designed clinical studies that combine early interventions (i.e., cognitive rehabilitation and supported employment in real-life competitive work settings) and long-term follow-up in civilian TBI-samples are warranted. As previous cognitive interventions have proven effective in reducing post-concussive complaints in the TBI population (31, 32, 35) and SE has been successfully applied in the Norwegian context to participants with mental illness (36), we developed a combined cognitive and vocational intervention to be tested in people with mild-to-moderate TBI who were still on sick leave 2 months post-injury due to persisting symptoms (37).

The aim of this study was thus to explore the effectiveness of a rehabilitation intervention with combined manualized cognitive rehabilitation efforts and SE in real-life competitive work-settings on employment participation following mild-to-moderate TBI. The main hypothesis was that those who received the study intervention would RTW sooner than patients receiving treatment as usual (TAU). Furthermore, it was expected that the intervention would result in increased work stability and productivity compared to TAU.

## Materials and Methods

### Study Design

We performed a single center pragmatic parallel-group RCT comparing the effectiveness of a combined cognitive and vocational intervention program to TAU on work participation in a civilian sample with mild-to-moderate TBI. The present study provides results from an interim analysis at 3- and 6-months, while subsequent publications will compare the effectiveness of the intervention and TAU on vocational and clinical outcomes up to 12 months after study inclusion.

### Study Population

Potentially eligible patients were referred from the Emergency department (ER), Neurosurgical department and GPs to an outpatient clinic at the Dept. of Physical Medicine and Rehabilitation (PM&R), Oslo University Hospital (OUH), Norway. The clinic provides specialized rehabilitation and follow-up services to patients with TBI. All patients referred to the clinic between July 2017 and April 2019 were eligible according to the following criteria: (1) diagnosed with mild-to-moderate TBI as assessed by a Glasgow Coma Scale (38) (GCS) score of 10–15, loss of consciousness (LOC) <24 h and posttraumatic amnesia (PTA) <7 days, (2) aged 18–60 years, (3) employed in a minimum 50% position at time of injury, and (4) sick listed 50% or more 8–12 weeks post-injury due to post-concussive symptoms as assessed with Rivermead Post Concussion Symptoms Questionnaire (RPQ) (39). The criteria for diagnosing mild TBI developed by the American Congress of Rehabilitation Medicine (40) were used to establish mild TBI, either according to patient records or while screening for eligibility. Individuals were excluded if they were active substance abusers, had severe pre-existing neurological or psychiatric conditions, and/or were unable to speak or read Norwegian.

### Procedures

Eligible patients who received oral and written information about the study were invited to participate by a medical doctor (MD) at the outpatient clinic, either during an initial consultation or later by phone. All patients who provided consent were contacted by phone to make an appointment for the baseline assessment where participants completed an assessment of self-reported symptoms and neurocognitive function. The following demographic characteristics were recorded: age, gender, education and marital status. Work-related information included occupation type (blue vs. white collar), occupation category, employment duration, full time position (yes vs. no), enterprise size, and percentage of sick listing at baseline. Clinical characteristics were also recorded and included time since injury (days), cause of injury, GCS score at time of injury or admission to hospital, duration of LOC and PTA, neuroimaging results, Abbreviated Injury Scale (AIS) head score, extracranial injuries (yes vs. no), admitted to hospital (yes vs. no), intoxication at time of injury (yes vs. no), and injured at the workplace (yes vs. no). The information was collected from medical records and self-report. Trained study personnel (clinical psychologist or MD) performed the baseline assessment at the outpatient clinic at PM&R.

### Randomization

A computer-generated permuted block randomization sequence was created by an independent statistician using randomized block sizes of 2, 4, 6, or 8 before initiating the study. Following baseline assessment, the participants were randomly allocated to the study intervention or TAU in a 1:1 ratio by an independent investigator who was not involved in the initial patient assessment. The intervention allocation was revealed to study personnel who contacted the participants to inform them about allocation and to arrange further follow-up. The nature of the intervention prevented blinding of participants or therapists providing the treatment. As randomization was performed after the baseline assessment, the study personnel performing these assessments were unaware of group allocation. Furthermore, outcome assessors performing the follow-up assessments were blinded. To prevent the participants from revealing group allocation, the assessors were instructed to inform the participants to not reveal the type of treatment they had received.

### Study Interventions

#### Treatment as Usual

TAU consisted of individual contacts and an educational group provided by a multidisciplinary team at PM&R, OUH. The specific treatment each participant received varied according to individual needs. An MD addressed physical problems related to the injury, while a neuropsychologist addressed psychological or cognitive complaints. An occupational therapist helped the patients structure their day and a social worker advised patients on issues relating to work, legal rights, and benefits. A physical therapist addressed vestibular symptoms and physical activity. In addition, the educational group entailed meeting 2 h once a week over a period of 4 weeks and addressed general information about mild-to-moderate TBI, common symptoms and problems in daily life, and advice regarding how to manage these.

#### Cognitive and Vocational Intervention

The combined cognitive and vocational intervention (CCT-SE) consisted of Compensatory Cognitive Training (CCT) and supported employment (SE). CCT is a manualized, group-based program to improve cognition and functioning in individuals who have sustained mild-to-moderate TBI (35) (the intervention manual is available from www.cogsmart.com). The intervention targets post-concussive and cognitive symptoms through psychoeducation and the implementation of compensatory strategies. CCT was provided in groups of 2–5 participants over a period of 10 weeks with one 2-h session each week. The intervention provides information about common symptoms that may occur after a TBI and strategies for dealing with fatigue, headache, sleep problems, and tension, in addition to specific strategies for cognitive problems. All participants were given a copy of the treatment manual and were assigned homework to increase generalizability of the learned strategies. Table 1 provides an overview of the cognitive domains targeted in the intervention and examples of strategies. The intervention manual was translated to Norwegian by researchers at PM&R, OUH, and Sunnaas Rehabilitation Hospital. To adapt the manual to a Norwegian civilian setting, we adjusted and down-scaled information about post-traumatic stress and injuries sustained in war settings. Before the intervention, the translated manual and an accompanying information leaflet was sent to The National Association for the Traumatically Injured (Personskadeforbundet LTN) who suggested minor changes. We performed a feasibility study prior to the RCT and found that the CCT program was acceptable within the Norwegian context (41).

**Table 1:** Topics covered in the CCT intervention.

**Session**	**Topic**	**Examples of strategies**
1	Course introduction and information about TBI	Finding a “home” for important personal items
2	Managing fatigue, sleep problems, headaches, and tension	Sleep hygiene and relaxation techniques
3	Organization and prospective memory	Time management and establishing routines
4	Organization and prospective memory (continued)	Calendar use and to-do lists
5	Attention and concentration	Paying attention during conversations
6	Learning and memory	Internal and external memory strategies
7	Learning and memory (continued)	Overlearning and name learning strategies
8	Planning and goal setting	Plan to meet goals and deadlines
9	Problem solving and cognitive flexibility	6-step problem solving method and self-monitoring
10	Skills integration, review, and next steps	Application of strategies to everyday life and progress toward goals

The vocational part of the intervention is based on supported employment (SE) principles (33). SE originates from research demonstrating that people with neuropsychiatric disabilities can perform complex work tasks and participate in paid work in the open labor market when appropriate level of support is provided (5, 33). The SE model consists of five stages: (1) Client engagement, (2) Vocational profiling, (3) Job finding, (4) Employer engagement, and (5) On and off the job support. Because all participants were employed at the time of injury, the main efforts in this study were on stages 1, 4, and 5. The first session focused on establishing a good working alliance, mapping the patient's resources, limitations and work tasks, and establishing common goals. Further follow-ups were tailored to the participants' needs and included work task adaptations, advice regarding assistive technology, learning new approaches, and training. The sessions included employers and other collaborators where appropriate. Participants received SE for a maximum of 6 months and the number of contacts between the participants and employment specialists and their content was recorded.

Both groups received standard Norwegian statutory sick leave follow-up in addition to the CCT-SE intervention or TAU.

### Treatment Fidelity and Adherence

Three employment specialists employed by the Norwegian Labor and Welfare Administration (NAV) delivered SE. They completed training in SE prior to the trial and received ongoing supervision during the trial. All sessions of the CCT intervention were provided at the TBI outpatient clinic at PM&R by a clinical psychologist or MD who received training by EWT prior to administering the program. A fidelity check list used to evaluate the therapist's adherence and competency in administering the CCT intervention was completed by senior researchers (ML and NA). The six checklist items which were chosen from a previous publication by Winter et al. (42) and a consensus in the project group were: 1- Explained content of each CCT session clearly; 2- Used appropriate pace and language; 3- Showed sensitivity to participants responses; 4- Responded clearly to participants questions; 5- Demonstrated overall fidelity to the CCT manual; 6- Explained next step of the CCT intervention. The rating levels were poor, good and excellent. Treatment fidelity was assessed for 30 (5%) CCT-sessions. The following items were on average rated as excellent: 2- appropriate pace and language; 3- sensitivity to participant's responses and 6- explained next steps of intervention. The remaining items were rated as good.

Attendance across the 10 sessions of the CCT-intervention was 99%, with only three participants missing a total of 6 sessions. Participants who were unable to attend sessions at the scheduled time (e.g., due to illness or other reasons) were rescheduled and given the opportunity to attend the session at a later time. When asked if they would recommend the CCT intervention to others with similar problems, 93% replied yes, 3.5% replied I don't know, and 3.5% replied no.

### Outcome Measures

The primary outcome was the proportion of participants who had returned to work (at any level). In addition, we assessed work percentage, stability, and productivity. The outcomes were collected at 3, 6, and 12 months following inclusion. As the 12 month follow-up is ongoing, this study reports work-related outcomes from the first two follow-ups.

The percentage of work participation was further divided into four categories relative to pre-injury employment grade (0 = not working at all; 1 = working <50%, 2 = working 50–79%, 3 = working 80–100%, i.e., full-time), describing the quantity of the work resumed at 3 and 6 months follow-up. Stable employment was defined as working at the same or increased level (%) as the previous follow-up time point (i.e., baseline to 3-months or 3 to 6 months follow-up), while unstable employment was defined as working at a decreased level (%) compared to the previous follow-up. Work productivity was operationalized by hours worked per week and whether there were accommodations at the workplace (yes/no). Participants were asked to describe the type of accommodations that were made. Number of hours worked per week was calculated by dividing 37.5 (i.e., standard time norm for full time work in Norway) by 100 and multiplying with work percentage relative to pre-injury work level at 3 and 6 months. All outcomes were collected by structured interviews.

### Statistical Methods

Data were analyzed with IBM SPSS Statistics for Windows v. 25 and Stata v. 16. Descriptive statistics are presented with mean and standard deviation (SD) or median and inter-quartile range (IQR) for continuous variables, and proportions and percentages or range for categorical variables. Between-group differences at each follow-up (3 and 6 months) were analyzed using independent samples *t*-tests for continuous and chi-square tests for categorical variables. Mixed effect models were fitted to all outcome variables to account for the repeated measures by patient. Continuous endpoints were analyzed using linear mixed models with random intercept and slope. Time and time-by-treatment interaction were fixed effects in all models. Based on the linear mixed model, we estimated mean values with 95% confidence intervals (CI) for the three time points (baseline, 3 months, and 6 months) for each treatment group. We also estimated the mean between group changes from baseline to 6 months. Dichotomous endpoints were analyzed using mixed effects logistic regression with treatment and time-by-treatment as fixed effects. Based on the mixed effects logistic regression we estimated risk differences with 95% CI from baseline to 3 and 6 months using the delta method. All analyses were done by intention to treat.

The overall sample size estimation was based on the primary outcome difference at 12-month follow-up, and is described elsewhere (37). The required sample size was estimated to 110 (i.e., 55 in each group). Based on a previous study (14), we estimated a loss to follow-up of 15%, requiring a total of 125 participants. However, loss to follow up was lower than expected and we concluded enrollment at 116 participants.

### Ethics

All subjects gave their informed consent for inclusion before they participated in the study. The study was conducted in accordance with the Declaration of Helsinki, and the protocol was approved by the Regional Committee for Medical Ethics in South-Eastern Norway (#2016/2038). The interventions involved no harm to the participants.

## Results

**Figure 1:**
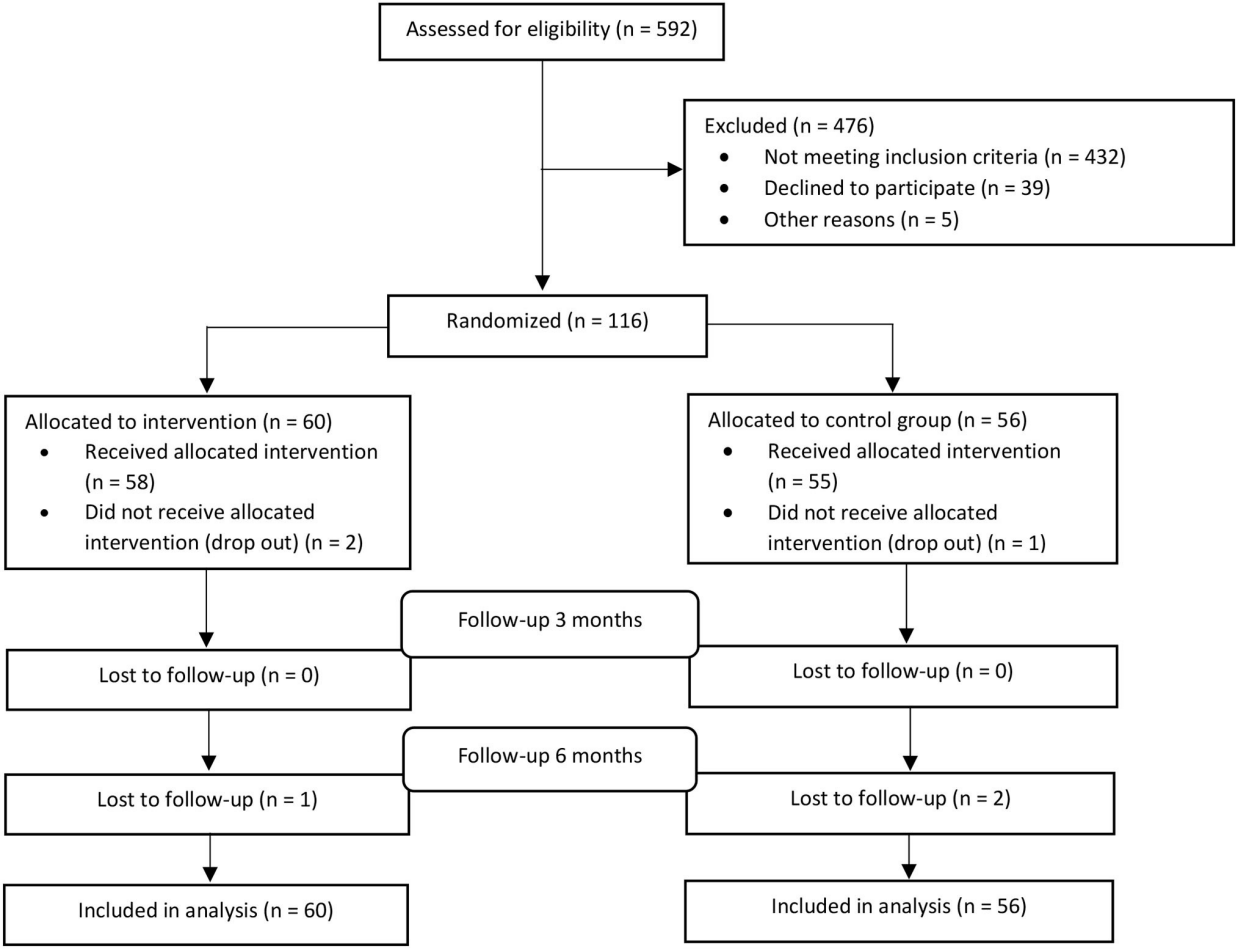
CONSORT flow chart.

A total of 116 patients were enrolled from July 2017 to April 2019 (see Figure 1). The main reasons for exclusion were time since injury (29%), age (17%) and sick leave <50% (10%). Only 3% of patients were excluded due to language barriers and 2% because of pre-injury comorbid conditions. Three participants dropped out after randomization, two before receiving any treatment, and one after completing one CCT session. Participants received the CCT-SE intervention or TAU from August 2017 until November 2019. There were no statistically significant differences between CCT-SE and TAU on baseline characteristics, with the exception of previous TBI and intoxication at the time of injury (see Table 2). Further analysis revealed that these variables were not associated with any of the outcomes, thus were not controlled for in the main analyses.

**Table 2:** Participant characteristics at baseline.

	**CCT-SE (*n* = 60)**	**TAU (*n* = 56)**
**Demographic information**		
Age, mean (SD)	41 (10)	44 (9)
Gender (female), n (%)	33 (55)	36 (64)
Education, mean (SD)	16 (2)	16 (3)
Marital status, n (%)		
Married/co-habitant	43 (72)	34 (61)
Divorced/separated/single	17 (28)	22 (39)
**Clinical information**		
Time since injury at inclusion (days), mean (SD)	77 (25)	68 (22)
Cause of injury, n (%) (*n* = 115)		
Fall	19 (32)	30 (54)
Transport	12 (20.5)	11 (20)
Blow to head	15 (25.5)	8 (14)
Sport	10 (17)	4 (7)
Violence	3 (5)	3 (5)
GCS, median (min-max) (*n* = 114)	15 (10–15)	15 (11–15)
LOC, n (%), (*n* = 115)		
None	31 (51.5)	30 (54.5)
<30 min	21 (35)	16 (29)
<24 h	1 (2)	2 (4)
Not registered	7 (11.5)	7 (12.5)
PTA, n (%), (*n* = 115)		
None	25 (42)	26 (47)
<1 h	18 (30)	17 (40)
<24 h	7 (11.5)	9 (16)
<7 days	0 (0)	2 (4)
Not registered	10 (16.5)	1 (2)
Trauma-related CT/MRI findings, n (%)		
Yes	11 (18)	16 (29)
No	45 (75)	35 (62)
No CT/MRI	4 (7)	5 (9)
AIS head score, n (%)		
Minor	34 (57)	25 (44.5)
Moderate	18 (30)	16 (28.5)
Serious	5 (8)	10 (18)
Severe	3 (5)	5 (9)
Extracranial injuries (yes), n (%)	28 (47)	25 (45)
Admitted to hospital (yes), n (%)	8 (13)	16 (28)
Intoxicated at time of injury (yes), n (%), (*n* = 115)	5 (9)	12 (21)
Injured at the workplace (yes), n (%), (*n* = 114)	9 (15)	7 (13)
**Work factors**		
Occupation type (white collar), n (%)	53 (88)	50 (89)
Occupation category, n (%)		
Military/Academic professions	30 (50)	28 (50)
Leaders	15 (25)	13 (23)
Office/Sales	10 (17)	9 (16)
Craft/Machine operators/Transportation/Cleaning	5 (8)	6 (11)
Employment duration (months), median (IQR), (*n* = 114)	54 (114)	42 (108)
Full time position (yes), n (%)	55 (92)	48 (86)
Enterprise size, n (%)		
Micro (1–9 employees)	4 (7)	5 (9)
Small (10–49 employees)	17 (28)	19 (34)
Medium (50–249 employees)	12 (20)	16 (28.5)
Large (>250 employees)	27 (45)	16 (28.5)
Sick listed, n (%)		
80–100%	48 (80)	46 (82)
50–79%	12 (20)	10 (18)

*CCT-SE, Compensatory Cognitive Training and Supported Employment; TAU, treatment as usual; GCS, Glasgow Coma Scale; LOC, loss of consciousness; PTA, post-traumatic amnesia; AIS, Abbreviated Injury Sale*.

The median duration of follow up in the TAU group was 155 days, and the median number of individual contacts per participant was 9. Of the 55 participants who received TAU, 100% were consulted by a MD, 50 (91%) received occupational therapy, 39 (71%) participated in the educational groups, 31 (56%) received physical therapy, 21 (38%) were referred to a neuropsychologist, and 20 (36%) received advice from a social worker.

The duration of the CCT-SE intervention is described in Materials and Methods section. Regarding SE, the total number of face-to-face meetings between the employment specialists and participants was 178 (on average three meetings per participant of which approximately one was at the work-place). The mean number of contacts per e-mail or telephone was 10 per participant.

### Proportion of Participants Returned to Work at 3 and 6 Months

**Figure 2:**
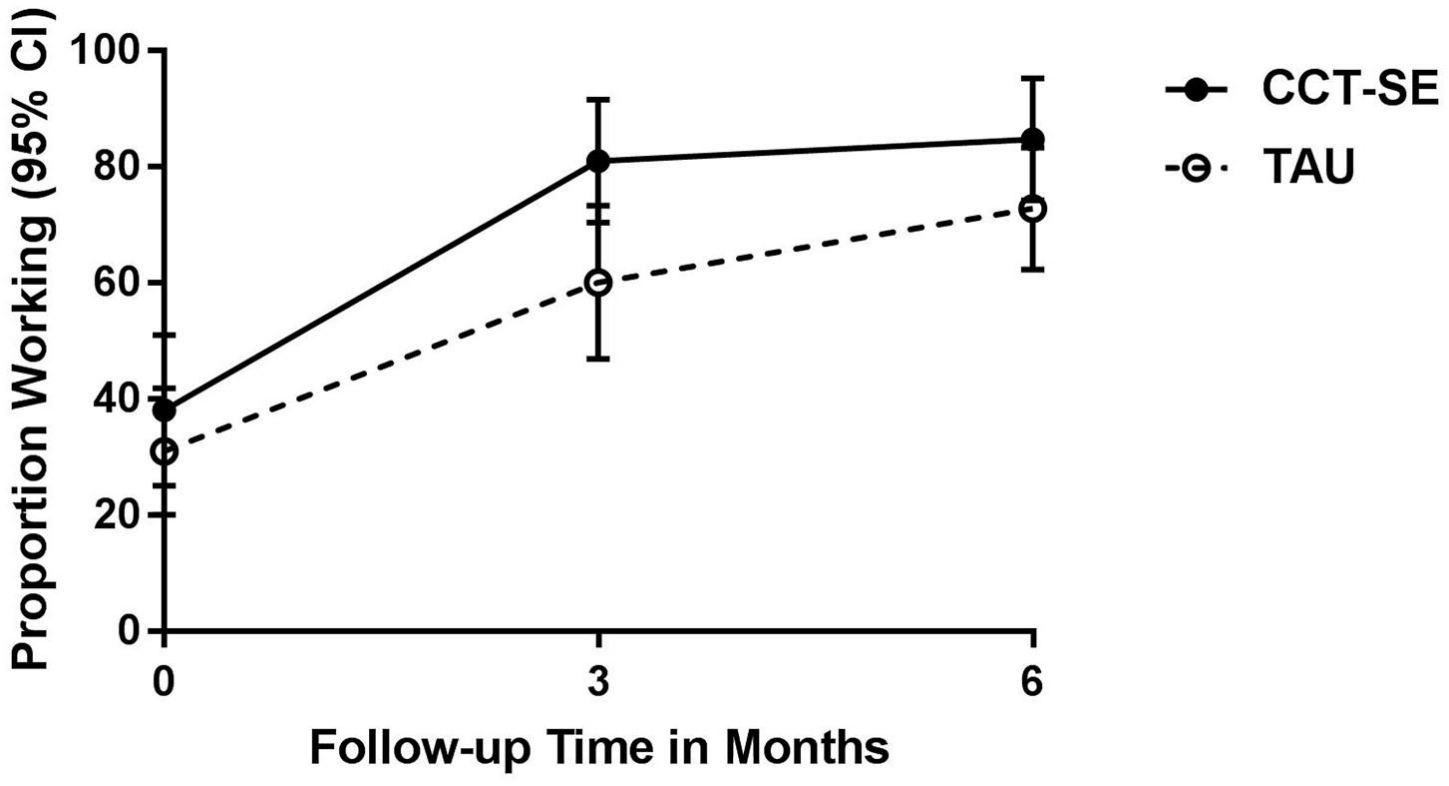
Estimated proportion of participants working at baseline, 3- and 6 months per treatment group from mixed effects logistic regression analyses. CCT-SE, Compensatory Cognitive Training and Supported Employment; TAU, treatment as usual.

At baseline, 40% in CCT-SE and 30% in TAU were working (at any level). At the 3 month follow-up, 81% in CCT-SE and 60% in TAU were working. At the 6 month follow-up, 84% in CCT-SE and 74% in TAU were working. There was a statistically significant higher proportion of participants working in the CCT-SE group compared to TAU at 3 months, but there was no difference between the groups at 6 months (see Figure 2). Mixed effects logistic regression analysis showed no between group difference regarding number of participants working from 3 to 6 months (Table 3). However, a statistically significant within group increase in number of participants working in both groups was observed.

**Table 3:** Results from mixed model analyses.

	**Baseline Proportion (95% CI)**	**3 months Proportion (95% CI)**	**6 months Proportion (95% CI)**	**Within group difference Baseline to 6 month, (95% CI), *p*-value**	**Between group difference (95% CI), *p*-value**
**Proportion working**					
CCT-SE	38.0 (25.1–51.0)	81.0 (70.4–91.5)	84.7 (74.2–95.2)	46.7 (32.9–60.5), *p* <0.001	4.8 (−13.3–23.0), *p* = 0.601
TAU	31.0 (20.1–41.8)	60.1 (46.8–73.3)	72.8 (62.3–83.3)	41.8 (30.0–53.6), *p* <0.001	
	**Baseline** **Mean (95% CI)**	**3 months** **Mean (95% CI)**	**6 months** **Mean (95% CI)**	**Mean within group change** **Baseline to 6 month,** ** (95% CI)**, ***p*****-value**	**Mean between group change (95% CI)**, ***p*****-value**
**Work percentage**					
CCT-SE	12.8 (8.2–17.4)	32.1 (26.2–38.0)	51.4 (41.9–60.9)	38.6 (29.6–47.7), p <0.001	2.0 (−11.0 to 15.1), *p* = 0.760
TAU	10.4 (5.6–15.2)	28.7 (22.6–34.8)	47.0 (37.2–56.8)	36.6 (27.2–46.0), p <0.001	
**Hours worked**					
CCT-SE	4.8 (3.1–6.5)	12.0 (9.8–14.3)	19.3 (15.7–22.8)	14.5 (11.1–17.9), *p* <0.001	0.76 (−4.1 to 5.7), *p* = 0.760
TAU	3.9 (2.1–5.7)	10.8 (8.5–13.1)	17.6 (14.0–21.3)	13.7 (10.2–17.3), *p* <0.001	

*Notes. CCT-SE, Compensatory Cognitive Training and Supported Employment; TAU, treatment as usual*.

### Work Percentage

**Figure 3:**
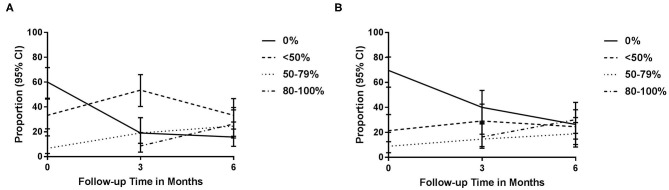
**(A)** Observed proportion of participants working 0%, <50%, 50–79%, and 80–100% at baseline, 3- and 6 months in the CCT-SE group; **(B)** observed proportion of participants working 0%, <50%, 50–79%, and 80–100% at baseline, 3- and 6 months in the TAU group. CCT-SE, Compensatory Cognitive Training and Supported Employment; TAU, treatment as usual.

Work percentage in CCT-SE and TAU from baseline to 3- and 6-month follow-up is shown in Figure 3. At baseline, 33% in CCT-SE and 21% in TAU were working below 50%, while 7% in CCT-SE and 9% in TAU were working 50% or more. At 3 months, 53% in CCT-SE and 29% in TAU were working below 50%, whereas 28% in CCT-SE and 31% in TAU were working 50% or more. At 6 months, 33% in CCT-SE and 24% in TAU worked below 50%; 51% in CCT-SE and 49% in TAU worked 50% or more. There was a statistically significant difference between the groups across the categories of work participation at 3 months, showing that for CCT-SE the highest proportion of work participation was in the category working <50% while for TAU the highest proportion was found in the not working at all category. The groups did not differ at 6 months. Using work percentage as a continuous variable, linear mixed model analyses showed no significant between group difference in work percentage from baseline to 3 and 6 months (Table 3). However, there was a statistically significant within group increase in work percentage.

### Hours Worked per Week

At baseline, the mean (SD) number of work hours per week in CCT-SE and TAU were 4.5 (6) and 4 (6), respectively. At 3 months, the CCT-SE group worked 13 (10) hours and TAU worked 11 (12) hours weekly. At 6 months, CCT-SE and TAU worked 19 (13) and 17 (15) hours per week, respectively. Mixed model analyses showed no between group differences in hours worked, but a statistically significant within group increase (Table 3).

### Work Stability

The majority of participants who were employed at 3 and 6 months were stably employed. In the CCT-SE group, three participants (2%) decreased their work percentage between baseline and 3 months, and three participants (2%) decreased their work percentage between 3 and 6 months. In the TAU group, two participants (1%) decreased their work percentage between baseline and 3 months, and four participants (2%) decreased their work percentage between 3 and 6 months. There were no statistically significant differences between the two groups regarding the proportion of unstably employed participants at 3 or 6 months. The number of unstably employed participants was too low to perform additional analyses.

### Work Place Accommodations

Among the 47 participants in the CCT-SE group who were working at 3 months, a total of 34 (72%) had accommodations made at the workplace, compared to 21 (64%) among the 33 who were working in the TAU group. At 6 months, 46 in CCT-SE were working with 25 (54%) having accommodations at the workplace compared to 19 (49%) of the 39 participants who were working in TAU. Accommodations included modified equipment (i.e., adjusted lighting, adapted computer screens, noise canceling head phones), flexibility with regard to working hours and location (i.e., opportunity to work from home, separate office, more breaks, limited traveling, exempt from night shifts), receiving help or allocating work tasks to someone else (i.e., hiring substitutes) and adjustment of work tasks (i.e., fewer tasks, exemption from stressful tasks and short deadlines). There were no statistically significant differences between the two groups regarding the proportion of participants who had accommodations at the workplace at 3 or 6 months.

## Discussion

The aim of this RCT was to compare the effectiveness of a combined cognitive and vocational intervention to multidisciplinary follow-up on employment participation in a sample of patients with mild-to-moderate TBI. Contrary to our hypotheses, we found no statistically significant differences in the measures of work participation between the CCT-SE and TAU groups from baseline to 6 months. Compared to TAU, a higher proportion of participants in the CCT-SE intervention group had returned to work at 3 months. In line with previous studies (28, 29), both groups had significant improvement regarding RTW, in addition to work percentage and hours worked during the 6-month study period.

The difference between the two groups regarding RTW proportion was seen during the first 3 months (11%, adjusted for group difference at baseline), i.e., while the participants were receiving both CCT and SE. Previous studies have recommended the use of compensatory cognitive strategies in rehabilitation following TBI (43). When facilitating RTW post-TBI, early supported employment could be applied and achieved when health care professionals, vocational counselors, and job coaches work together (26, 44, 45). Our finding is in contrast to the previously mentioned study by Scheenen and colleagues finding no between-group differences regarding RTW at 3 months follow-up (28). However, the results may not be directly comparable as Scheenen et al. provided less complex interventions and assessed full RTW. However, the proportion of participants working doubled in both groups, and the observed difference was no longer present at the 6-month follow-up. This may indicate that the TAU group, although taking longer to RTW, continued to improve over time and eventually reached the same level. Although the TAU group only received statutory sickness absence follow-up, and not SE at the workplaces, the multidisciplinary follow-up may have positively influenced the patients' conditions and frequency of RTW. As we did not include a no-treatment control group, we were not able to establish the influence of the natural course of recovery or the isolated contribution of CCT-SE, as the TAU group also received multidisciplinary treatment, although less specific and not manualized.

The TAU group received individual follow-up by a multidisciplinary team and limited group-based education about common symptoms and problems, while the CCT-SE group received individualized work support and a combination of psychoeducation about TBI, strategies to manage common symptoms and compensatory cognitive strategies. As such, both groups received rather extensive follow-up, although the content of the two treatments differed. The effect of the multidisciplinary follow-up was assessed in a previous publication and found to reduce number of post-concussive symptoms, but did not improve RTW (29). Previous studies have reported that the CCT program can reduce subjective complaints and improve neurocognitive function in veterans with mild-to-moderate TBI (31, 32, 35), while SE has been shown to significantly improve work outcomes in individuals with mental health issues in the Norwegian context (36).

Improving early RTW rates post-TBI is of personal and socioeconomic importance. However, the literature shows that individuals who RTW may experience continued difficulties affecting work stability and productivity (10, 12). Results from this study showed that the majority of participants in both groups were stably employed between baseline, 3, and 6 months. Furthermore, there was a significant increase in hours worked from baseline to 6 months. Thus, the findings did not suggest that either CCT-SE or TAU had a negative impact on objective measures of stability and productivity, and that neither treatment group actually contributed to premature RTW with negative effect.

Although we did not find a significant between-group difference regarding work percentage in the overall model, a higher proportion of individuals in the CCT-SE group had returned to part-time work at 3 months. There was also a non-significant higher proportion of individuals with accommodations at the work-place in the CCT-SE group at the 3 month follow-up. SE aims to provide individually adapted work-support, including advice regarding adapting work tasks and assistive technology to increase the chance of successful RTW, while the CCT intervention provides psychoeducation, stress reduction techniques and compensatory strategies for cognitive complaints. Moreover, the therapists providing CCT and SE worked in close collaboration, thereby increasing the chance of identifying specific issues and implementation of individualized strategies at the work place. These components may have positively influenced the participants' RTW process at an early stage

In general, the proportion of participants that returned to work at 6 months was high in both groups. Still, 16% of the participants in the intervention group and 26% in the TAU group had not returned to work at 6 months. Furthermore, only 35% of the participants on average were working between 80 and 100%, indicating that many participants were still working at a reduced level compared to before their injury. The proportion of participants who did not RTW is comparable to the study by de Koning et al. (19) who included a sample with comparable patient characteristics. Norway is characterized by high job security, low unemployment, and a comprehensive welfare system where patients receive a full salary the first year of sick-leave. However, the rate of sickness absence is among the highest in Europe, and the mentioned sickness benefits may reduce the impact of interventions aimed to improve RTW (46).

### Strengths and Limitations

The majority of the participants had sustained a mTBI and were recruited based on experiencing persistent complaints 2 months following injury. Furthermore, they were employed in a minimum 50% position at the time of injury. More women than men were recruited to this study, which diverges from epidemiological TBI studies. As such, the results may not be generalizable to all patients with TBI, including individuals who are unemployed at the time of injury. Additionally, the results should be generalized with caution with regard to gender differences. However, the study sample was civilian as opposed to veterans, generalizing the results beyond the military context. The study also extends existing data to a sample derived within a Scandinavian welfare system.

To avoid interference from the researchers or therapists providing the intervention or TAU, the required medical sick-leave certificates were completed by the participants' GPs. Information about sick-listing, percentage of sick leave and number of hours worked per week was self-reported by the participants at each follow-up. Self-reported work status has been found to be reliable in other patient populations (47) and we regard the participants' self-reports as valid as they regularly visited GPs for sick-leave certifications.

The study was performed in Norway, a welfare state with long-term sickness benefits. TAU, in this context, encompassed individualized multidisciplinary follow-up provided by experienced therapists which can be considered a specialized version of usual care. The level of treatment provided in the TAU group represents a University hospital service delivery in the capital of Norway, which might not be representative for patients treated in other hospitals or geographical regions, or countries with different organization of health care. This may have influenced the results of the study. Additionally, control groups receiving CCT or SE only might have made it possible to tease apart the effect of specific components of the combined intervention. Due to a limited number of TBIs in our region (6), we were unable to design the study with more than one control group.

Moreover, as an interim report, we are unable to make a conclusive statement about the overall primary outcome (i.e., the proportions of RTW) at 12-month follow up. Nevertheless, this interim report provides the outcomes from the first two planned follow ups, independent from the final 12-month follow-up data. As the trial continues, we plan to provide a final report regarding the long-term RTW parameters once the final data set becomes available.

This is the first study conducted in close collaboration between hospital staff, job coaches from the Norwegian Labor and Welfare organization and the Work Research Institute (i.e., trans-sectorial collaboration). The outcomes were selected based on recommendations from a previous systematic review on vocational interventions after TBI (34) thus describing a broader range of employment outcome. Furthermore, findings from a process evaluation across sectors will be published in a subsequent paper.

## Data Availability Statement

The raw data supporting the conclusions of this article will be made available by the authors, without undue reservation.

## Ethics Statement

This study was reviewed and approved by the Regional Committees for Medical and Health Research Ethics (REC), South East Norway. The patients/participants provided their written informed consent to participate in this study. The trial was registered at the US National Institutes of Health (ClinicalTrials.gov) #NCT03092713.

## Author Contributions

EH, SF, ML, HE, and NA: conceptualization. EH, ML, HE, CR, ET, JL, and NA: methodology. EH, ML, CB, and NA: formal analysis. EH, SF, ML, KS, TH, and NA: investigation. EH, SF, ML, KS, TH, TN, and NA: data curation. EH, ML, and NA: writing original draft preparation. EH, SF, ML, HE, KS, TH, CB, CR, TN, HS, ET, JL, and NA: writing, review and editing. EH, CB, NA, and ML: visualization. NA, ML, CR, TN, HE, and HS: supervision. NA, ML, CR, and HE: project administration. NA: funding acquisition. All authors have contributed substantially to the work reported, critically revised the paper, read and approved the final manuscript.

## Conflict of Interest

The authors declare that the research was conducted in the absence of any commercial or financial relationships that could be construed as a potential conflict of interest.
